# Multiple heavy metal tolerance and removal by an earthworm gut fungus *Trichoderma brevicompactum* QYCD-6

**DOI:** 10.1038/s41598-020-63813-y

**Published:** 2020-04-24

**Authors:** Ding Zhang, Caiping Yin, Naeem Abbas, Zhenchuan Mao, Yinglao Zhang

**Affiliations:** 1 School of Life Sciences, Anhui Agricultural University, Hefei, 230036 P.R. China; 2 Institute of Vegetables and Flowers, Chinese Academy of Agricultural Sciences, Beijing, 100081 China

**Keywords:** Applied microbiology, Pollution remediation

## Abstract

Fungal bioremediation is a promising approach to remove heavy-metal from contaminated water. Present study examined the ability of an earthworm gut fungus *Trichoderma brevicompactum* QYCD-6 to tolerate and remove both individual and multi-metals. The minimum inhibitory concentration (MIC) of heavy metals [Cu(II), Cr(VI), Cd(II) and Zn(II)] against the fungus was ranged 150–200 mg L^−1^ on composite medium, and MIC of Pb(II) was the highest with 1600 mg L^−1^ on potato dextrose (PD) medium. The Pb(II) presented the highest metal removal rate (97.5%) which mostly dependent on bioaccumulation with 80.0%, and synchronized with max biomass (6.13 g L^−1^) in PD medium. However, on the composite medium, the highest removal rate was observed for Cu(II) (64.5%). Cellular changes in fungus were reflected by TEM analysis. FTIR and solid-state NMR analyses indicated the involvement of different functional groups (amino, carbonyl, hydroxyl, *et al*.) in metallic biosorption. These results established that the earthworm-associated *T. brevicompactum* QYCD-6 was a promising fungus for the remediation of heavy-metal wastewater.

## Introduction

Environmental pollution by heavy metals (such as Cr(VI), Cu(II), Cd(II), Pb(II) and Zn(II)), is widespread owing to urbanization, industrialization and agricultural practices^1,2^. As multi-metals are non-biodegradable, they are constantly present in the environment and bio-accumulate in the tissues of plants, animals and humans, resulting in bio-magnification in the food chain. Biomagnified heavy metals lead to health disorders, such as osteomalacia, birth defects, and health hazards to the liver, kidneys, nervous and gastrointestinal systems^3,4^. Toxic heavy metals are traditionally treated by physicochemical techniques, such as precipitation, membrane filtration, adsorption, and chemical oxidation-reduction^5^. However, these methods are generally costly and ineffective when the metallic concentrations are low^1^. The use of microorganisms to bioremediate is a cheaper and environmental friendly alternative approach to remove heavy metals from environment^6–8^.

Earthworms have the ability to accumulate heavy metals, including Cu, Cd, Pb and Zn^9^. In general, metallic accumulation by earthworm occurs through two pathways, which include absorption following dermal contact and adsorption through the intestinal tissues^10–12^. It has been reported that the gut microbiota of other animals were found to exhibit strong metals tolerance and binding abilities^13,14^. So, it could be hypothesized that the intestinal microorganisms of earthworms have the ability of heavy metal tolerance and removal.

In the course of our ongoing efforts to screen potential strain for metal removal from the gut microbiota of earthworm, we found that intestinal fungus *Trichoderma brevicompactum* QYCD-6 of *Pheretima tschiliensis* exhibited good potential for heavy metal tolerance. Here, we reported the details of the ability and mechanisms of tolerance and removal of the individual heavy metal as well as multiple metals by *T. brevicompactum* QYCD-6. To our knowledge, the intestinal microorganisms of earthworms have not been reported for metal removal and hence this report would be of great importance in the bioremediation of heavy metals from polluted places.

## Materials and methods

### Isolation and identification of fungus from *P. tschiliensis*

The fungal strain was isolated according to the methods detailed previously with trivial changes^15^. The *P. tschiliensis* was collected from the campus of Anhui Agricultural University, Hefei China (31°53′29″N; 117°14′10″E). The earthworm was transported to the laboratory and starved for a day. The sample was sterilized in 75% ethanol for 3 min, followed by rinsing thrice with sterilized water, and then the earthworm was degutted using sterile scalpel and tweezer. The gut was homogenized, and dilution series (10^−1^, 10^−2^, and 10^−3^) were spread-plated on PDA (20 g L^−1^ glucose, 20 g L^−1^ potato, 20 g L^−1^ agar) plates supplemented with streptomycin sulfate (100 mg L^−1^). The plates were incubated at 28 °C for 4 days for fungus growth and any fungal colonies that formed were subcultured on new PDA medium to obtain pure cultures.

Morphological characterization and molecular identification (based on the DNA sequence of the ITS region using ITS1 and ITS4 primers after PCR amplification) were implemented to identify the strain.

### Heavy metal tolerance and growth conditions for fungus

Heavy metal reserve (1000 mg L^−1^) was prepared by dissolving respective metallic salts (CuSO_4_, CdSO_4_.8/3H_2_O, K_2_Cr_2_O_7_, Pb(NO_3_)_2_ and ZnSO_4_.7H_2_O) in double distilled water, and diluted to appropriate concentration before the experiments^16^. Tolerance to metals [Cu(II), Cr(VI), Cd(II), Zn(II) and Pb(II)] by *T. brevicompactum* QYCD-6 was determined according to the MIC of metals. The fungal strain was grown in PD (10 g L^−1^ dextrose, 20 g L^−1^ potato) and composite media (MgSO_4_.7H_2_O, 0.1 g L^−1^; NH_4_NO_3_, 0.5 g L^−1^; NaCl, 1 g L^−1^; K_2_HPO_4_, 0.5 g L^−1^; Yeast extract, 2.5 g L^−1^; Glucose, 10 g L^−1^) having different metallic concentrations and pH was adjusted to 7.0. Metal-containing media were inoculated with fungal spore suspension (10^6^ spores mL^−1^) at 28 °C on rotary shakers at 150 rpm.

### Effect of heavy metal on growth kinetics and metal removal mechanism

According to the methods reported by Gola *et al*.^16^, with some modifications, the effect of heavy metals on the growth kinetics of fungus was explored in presence of 50 mg L^−1^ individual metals and in multi metal mixture [10 mg L^−1^ Cu(II), Cr(VI), Cd(II), Pb(II) and Zn(II) each]. 1 mL *T. brevicompactum* QYCD-6 spore suspension (10^6^ spores mL^−1^) was inoculated in 100 mL composite media, except PD medium for 50 mg L^−1^ Pb. Flasks were removed every 24 h and samples were analyzed for pH profile, glucose concentration, residual metal concentration and biomass production. Fungal biomass was filtered by Whatman No.1 paper, then the filter paper was fully dried at 60 °C to a constant weight and gravimetric method was used to determine the biomass.

### Effect of initial heavy metal concentration

The influence of different concentrations (30, 50, 100 mg L^−1^) of individual metals [Cu(II), Cr(VI), Cd(II), Pb(II) and Zn(II)] and metal mixture on fungal biomass, metal removal and specific metal ion uptake of *T. brevicompactum* QYCD-6 was detected by inoculating composite media with 1% spore suspension except PD medium for Pb(II) analysis.

### Measurement of residual heavy metal concentration

Ten mL of the samples from culture fluid were removed aseptically and centrifuged at 4,000 rpm for 10 min. The supernatant was filtered through a 0.45 μm microporous filter with a PTFE syringe. Individual ions were analyzed by flame atomic absorption spectrophotometer (FAAS, Analytik Jena, ZEEnit 700 P, Germany) and the multi-metal mixture was determined by inductively coupled plasma-atomic emission spectrometry (Thermo Scientific iCAP 6300 Duo).

### Estimation of glucose content

Glucose content was analyzed using the DNS method as described in literatures^16,17^. The DNS reagent was prepared by dissolving 6.3 g 3, 5-dinitro salicylic acid, 182 g of potassium sodium tartrate, and 262 mL of 2 M NaOH in 500 mL deionized water at 50 °C. Then 5 g of phenol and 5 g of sodium sulfite were added in the solution, and cooled down to room temperature to give the DNS reagent. The calibration curve was constructed with D-glucose (Sigma-Aldrich) solutions at different concentrations. The analyses were made with 1 mL sample in 3 mL of DNS, which were heated at 100 °C for 5 min. After cooling, the sample was evaluated at 540 nm UV–vis.

### Fourier transformed infrared (FTIR) spectral analysis

The functional groups responsible for heavy metal uptake on *T. brevicompactum* QYCD-6 were investigated by FTIR. Fungal biomass from the flask containing 50 mg L^−1^ heavy metal (individual metal or metal mix) and control was collected and lyophilized, and then 1 mg fully lyophilized biomass was mixed with 150 mg KBr. Infrared spectra were recorded on a Nicolette is50 (Thermo Fisher Scientific, USA) FTIR spectrometer.

### Transmission electron microscopy (TEM) analysis

TEM analysis was performed according to the methods described previously with small changes^18^. Fungal biomass was obtained by centrifugation at 10000 rpm for 5 min, then washed with phosphate buffer saline (pH 6.8) and subsequently fixed in 2% paraformaldehyde and 2.5% glutaraldehyde for 12 h. Subsequently, the fungal cells were postfixed using 2% osmium tetroxide for 1 h. The fixed cells were dehydrated in an acetone gradient series and embedded in epon-araldite. Ultra-thin sections with thickness of 60–80 nm were cut by an ultramicrotome (Leica EM UC7). The sections were stained with lead citrate and alcoholic uranyl acetate for 10 min, before detecting the copper grids in a TEM (Hitachi-HT7700).

### Solid-state nuclear magnetic resonance (NMR) analysis

The solid-state ^13^C cross polarization/total sideband suppression (CP/TOSS) NMR spectra were recorded on a Bruker AVANCE III 400 WB spectrometer equipped with a 4 mm standard bore CP/TOSS probe head whose X channel was tuned to 100.62 MHz, using a magnetic field of 9.39 T at 297 K. The dried and finely powdered samples were packed in the ZrO2 rotor closed with Kel-F cap which were spun at 5 kHz rate. The experiments were conducted at a contact time of 2 ms. 4096 scans in total were recorded with 1 s recycle delay for each sample.

### Statistical analysis

All experiments were performed in triplicate. A least significant difference (LSD) test with a confidence interval of 95% was used to compare the means.

## Results and discussion

### Identification of the fungus

The morphological characteristics of the fungus were observed in the PDA medium. The isolated QYCD-6 mycelium grew rapidly at 28 °C in the darkness. The aerial mycelia were white to green and colorless on the reverse side of the plate. Phialides were densely clustered on hyphae. Conidia were gray-green, smooth and elliptical. The cultural morphology of the isolates was consistent with that of *Trichoderma* species^19,20^. Phylogenetic taxonomy with a sequence alignment of ITS rDNA of the fungus was performed with MEGA 5.0 software. The phylogenetic tree (Fig. S1) indicated that the fungus was closely related to *Trichoderma brevicompactum* ACCC32847 (MF780855.1), with the ITS sequence similarity of 99.7%. Combined with the morphological characteristics, the fungus was identified as *T. brevicompactum*.

### Minimum inhibitory concentration

Minimum inhibitory concentration (MIC) of different heavy metals [Cu(II), Cr(VI), Cd(II), Pb(II) and Zn(II)] for *T. brevicompactum* QYCD-6 ranged 250–1600 mg L^−1^ on PD media with highest MIC recorded for Pb(II) followed by Zn(II) as summarized in Table 1. The MIC of heavy metal [Cu(II), Cr(VI), Cd(II) and Zn(II)] was relatively low with ranged 150–200 mg L^−1^ on composite media. Although the fungus did not exhibit high MIC against tested metals as described by recently reports of *Aspergillus lentulus*^21^ and *Paraphaeosphaeria* sp. SR46^22^, the strain displayed higher MIC against the heavy metal [Cu(II), Cr(VI) and Cd(II)] than that of *Beauveria bassiana* with 100 mg L^−1^ on composite media, which suggests that the *T. brevicompactum* QYCD-6 will survive under the moderately contaminated sites encountered in the environment^16^.

**Table 1:** MIC of heavy metal for *T. brevicompactum* QYCD-6 (mg· L^−1^).

Metal	PD media	Composite media
Cu(II)	300	200
Cr(VI)	300	150
Cd(II)	250	150
Pb(II)	1600	NT^a^
Zn(II)	450	150

^a^Not tested.

### Effect of heavy metal on growth kinetics and metal removal mechanism

**Figure 1:**
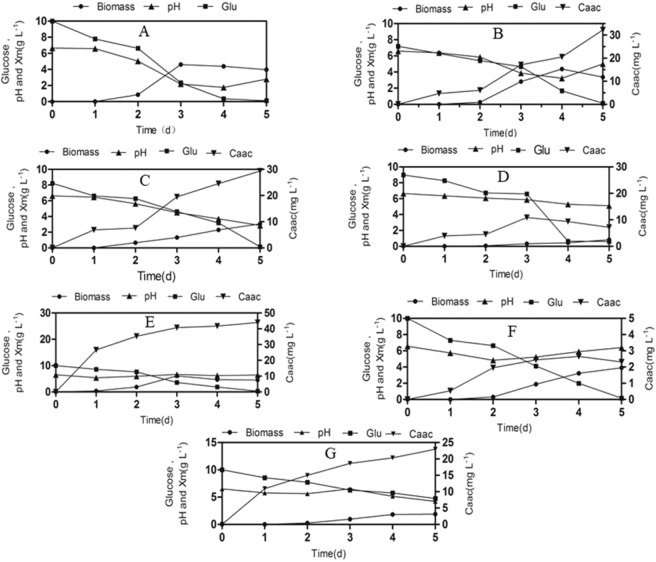
Growth kinetic and removal of heavy metal by *Trichoderma brevicompactum* QYCD-6 with (**A**) in absence of metal; (**B**) at 50 mg L^−1^ of Cu; (**C**) at 50 mg L^−1^ of Cr; (**D**) at 50 mg L^−1^ of Cd; (**E**) at 50 mg L^−1^ of Pb; (**F**) at 50 mg L^−1^ of Zn; (**G**) at 50 mg L^−1^ of Multi Metal Mix. The fungus was grown in composite media except that of 50 mg L^−1^ of Pb (in PD medium).

The changes in pH, glucose consumption and produced biomass of *T. brevicompactum* QYCD-6 with or without individual heavy metal and metal mixture in the media were shown in Fig. 1. A decrease in the pH (6.6–4.8) was detected during the lag phase of the fungus in the presence and absence of heavy metal. It was perceived that the lag phase was relatively short and limited to 1 day in the presence of Pb and absence of heavy metal, as compared to 2 days in the presence of other metals. The extension of the lag phase indicated the adaptation of the fungal spore to the environment, corresponding to the slow rate of glucose utilization for fungus with metal (Cu(II), Cr(VI), Cd(II), Zn(II), multi metal mix) as displayed in the Fig. 1. A similar increment in the lag phase with metals was observed for *A. lentulus*^23^. During 5 d, the heavy metal Pb(II) presented the highest metal removal rate (97.5%) and synchronized with maximum biomass (6.13 g L^−1^) on the PD medium. However, on the composite medium, the highest removal rate of metal was Cu(II) (64.5%), followed by multi metal mixture (45.9%), Cr(VI) (24.8%), Cr(VI) (24.8%), Cd(II) (16.8%) and Zn(II) (4.6%), and the maximum fungal biomass was appeared in absence of any metal (4.61 g L^−1^ in 3d), which decreased to a certain amount in presence of Cu(II) (4.38 g L^−1^), Zn(II) (3.91 g L^−1^), Cr(VI) (3.05 g L^−1^), multi metal mixture (1.90 g L^−1^), and Cd(II) (0.83 g L^−1^). Hence, all the tested heavy metals except Pb(II) induced growth inhibition to some extent. Nongmaithem *et al*. (2016) also discovered that under single metal exposure, Cd(II) caused a higher reduction in biomass growth of *Trichoderma* sp. MT-4, and UBT-18 as compared to Ni(II)^24^. Although metal Zn(II) supported moderate fungal biomass production, the corresponding metal removal could not be acquired. This indicated that the removal mechanism of the Zn(II) metal might not be directly paralleled with the increment of fungal biomass by surface area. Evidently higher metal removal (64.5%) was observed for Cu(II), which supported moderate fungal biomass production (4.38 g L^−1^).

**Figure 2:**
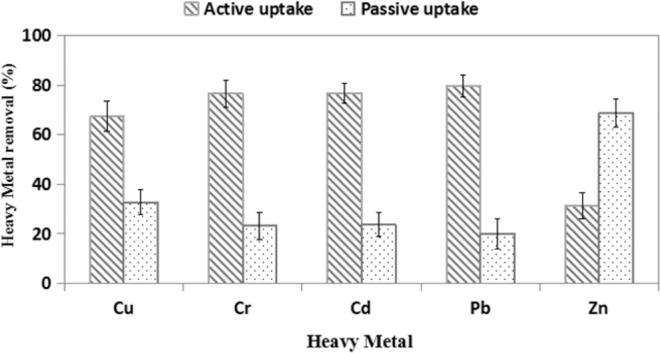
Passive and active heavy metal uptake by *T. brevicompactum* QYCD-6 in multi metal mixture.

In order to clarify the removal mechanism of multi metal, the passive and active uptake of heavy metal was examined (Fig. 2). It was observed that a significant portion of Zn(II) and Cu(II) was removed by the biosorption mechanism. Removal of Zn(II) up to 68.7% and 32.7% Cu(II) was owing to biosorption. However, uptake of Pb(II) was mostly due to bioaccumulation (80.0%) in comparison with the biosorption (20.0%), followed by Cd(II) and Cr(VI). Therefore, it appeared that the removal of heavy metals has being influenced by both bioaccumulation and biosorption simultaneously^16^.

### Effect of initial metal ion concentration on uptake capacities

Heavy metal uptake capacities of *T. brevicompactum* QYCD-6 at the different initial metal concentration in the media were shown in Table 2. When the initial metallic concentration was increased from 30 mg L^−1^ to 100 mg L^−1^, the fungal biomass was decreased for all heavy metal except Pb. The maximum reduction in fungal biomass appeared in presence of Cd(II) with a 97.2% of decrease followed by the multi-metal with 87.5%, Cr (VI) 59.6%, Zn(II) 56.4%, Cu(II) 55.7%, indicating metal Cd(II) was more toxic in comparison with other heavy metals. A decrease in biomass coupled with an increase in the initial heavy metal concentration was also observed for other fungal isolates. For example, Cd-induced decline of biomass for *A. foetidus*^25^ and *A. aculeatus*^26^ was observed over a concentration of 5 to 25 mM and 5 to 200 mg L^−1^, respectively. Pb(II) displayed an interesting behavior. The biomass decreased initially from 5.12 g L^−1^ at 30 mg L^−1^ to 4.64 g L^−1^ at 50 mg L^−1^ and then increased to 6.05 g L^−1^ for 100 mg L^−1^.

**Table 2:** Bioaccumulation of Cu(II), Cr(VI), Cd(II), Pb(II), Zn(II) and multi metal mixture by *T. brevicompactum* QYCD-6 (at 30 °C, 150 rpm and 120 h).

Metal	Co (mg L^−1^)	Xm (g L^−1^)	Ct (mg L^−1^)	Qm (mg g^−1^)	Uptake yield (%)
Cu(II)	30	3.88	18.12	4.67	60.40
	50	3.14	32.23	10.26	64.46
	100	1.72	60.31	35.06	60.31
Cr(VI)	30	4.98	6.94	1.39	23.13
	50	3.05	12.40	4.07	24.80
	100	2.01	31.83	15.84	31.83
Cd(II)	30	1.08	6.04	5.59	20.13
	50	0.83	8.42	10.14	16.84
	100	0.22	8.45	38.41	8.45
Pb(II)	30	5.12	28.80	5.63	96.00
	50	4.64	48.77	10.51	97.54
	100	6.05	81.11	13.40	81.11
Zn(II)	30	4.13	1.33	0.32	4.43
	50	3.91	2.31	0.59	4.62
	100	1.80	2.47	1.37	2.47
Multi metal	30	3.11	11.80	3.79	39.30
	50	1.89	22.95	12.14	45.90
	100	0.39	48.57	124.53	48.57

X: Dried biomass; Co: Initial metal ion concentrations; Ct: metal removal after 5 days; Qm: Specific metal ion uptake determined as the amount of metal per unit of dry biomass.

Generally, the specific heavy metal ion uptake of the fungus increased with the increment in the initial heavy metal concentration. The maximum individual metal ion uptake was calculated as 38.41 mg g^−1^ for Cd(II), followed by 35.06 mg g^−1^ for Cu(II), 15.84 mg g^−1^ for Cr(VI), 13.40 mg g^−1^ for Pb(II) and 1.37 mg g^−1^ for Zn(II) at 100 mg L^−1^ initial metallic concentration. As for multi metal mixture, the maximum specific uptake of 124.53 mg g^−1^ was detected at 100 mg L^−1^ concentration. The increment in specific heavy metal uptake was also examined with the fungi *A. foetidus*^25^ and *T. asperellum* TS141^27^ when the initial metallic concentration was increased. Chen also reported an increase in the concentration of Pb(II) from 50 ppm to 200 ppm resulted in an improved metal uptake for *Simplicillium chinense* and *T. asperellum*^28^.

The above results illustrated that *T. brevicompactum* QYCD-6 could be applied for the treatment of wastewater polluted with multiple heavy metals. Although the adsorption capacity of fungus was not as good as that of nanocomposite material^29,30^, the fungus was highly appropriate for remediation of irrigational waters with a low concentration of multiple heavy metals^16^. In addition, the strain could be used for remediation of industrial sewages, which release selected metals.

### TEM analysis on ultrastructural changes of* T. brevicompactum* QYCD-6

**Figure 3:**
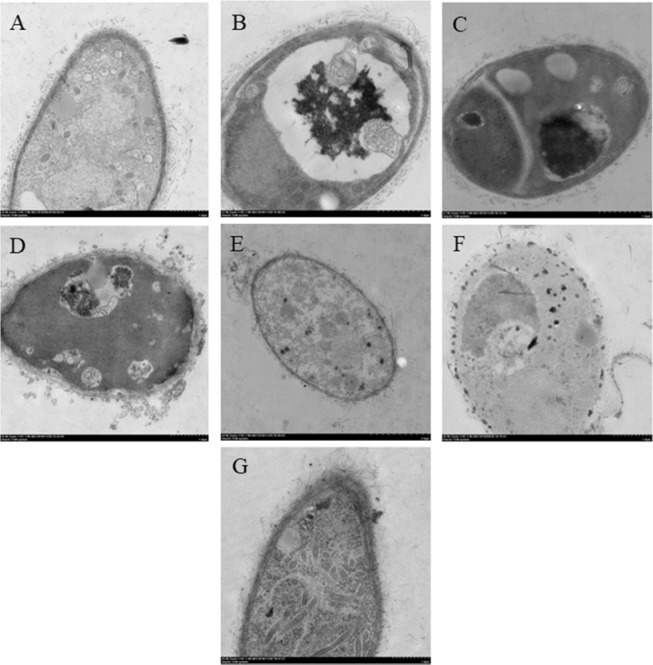
TEM of *T. brevicompactum* QYCD-6. (**A**) In absence of metal; (**B**) at 50 mg L^−1^ of Cu (**C**) at 50 mg L^−1^ of Cr; (**D**) at 50 mg L^−1^ of Cd; (**E**) at 50 mg L^−1^ of Pb; (**F**) at 50 mg L^−1^ of Zn and (**G**) at 50 mg L^−1^ of Multi Metal Mix.

The ultrastructural changes of *T. brevicompactum* QYCD-6 aroused by individual heavy metal as well as the metal mixture were detected by using TEM (Fig. 3). Defined cell membrane and organized distribution of cytoplasm with the intact cells under the TEM micrograph were displayed in Fig. 3A. The ultrastructural changes with discernible dark spots in fungus were observed in the presence of heavy metals (Fig. 3B–G). Minimum damage to the cell was shown by Pb(II) with few visible dark spots emerge inside the fungal cell. More damage to the cellular structure with a dense dark area was presented both inside the cell as well as in membrane by Zn(II) and multiple heavy metals, suggesting the involvement of both bioaccumulation and biosorption. This was correlated to the results gained with their FTIR analysis. Similar dense and dark areas were observed both in the cell membranes and inside the *B. bassiana* cell grown in the presence of Cr(VI) and Zn(II)^16^. While as for Cr(VI) and Cd(II), dark spots have occupied many areas inside the fungal cell, indicating their major bioaccumulation mechanism. Noteworthy was the bioaccumulation of more amorphous electron-dense spots in the vacuoles of Cu(II)-treated fungus, which was similar to that of *Humicola lutea* 103^31^.

### FTIR analysis on functional groups of fungal biomasses

The characteristic infrared peaks of *T. brevicompactum* QYCD-6 before and after metal absorption were acquired within the range of 450–4000 cm^−1^. As shown in Table 3, the involved functional groups of fungal biomasses included amino, hydroxyl, carbonyl, phosphoryl, nitro, and other groups. The specific treatment with Cu, Cr, Cd, Pb, Zn and metal mix gave rise to the drift of the peak at 3424.0 cm^−1^ (representing N-H and O-H groups), 1652.1 cm^−1^ (N-H deformation of amide I band and C=O stretching), 1552.3 cm^−1^ (C-N stretching in -CO-NH- and N-H bending in amide II, except for Pb), 1400.9 cm^−1^ (carboxylate group), 1034.3 cm^−1^ (C=C, C-C, C-O-P and C-O-C groups of saccharides, except for Zn) and 562.6 cm^−1^ (disulphide groups and nitro compounds) (Table 3; Supplementary Fig. S2). Interactions between sulfide, phosphate, and metals might have happened on account of the shift at 1074.4 cm^−1^ (except Cr) and 1154.7 cm^−1^. Additional peaks were also discovered at approximately 2853 cm^−1^ (C-H symmetric stretching) for Cu and Pb. Both Cu- and Cr-treated cells appeared new peaks at approximately 2426 (P-H stretching) (Table 3; Supplementary Fig. S2). Only one new peak of 1744.5 (C=O stretching of ester) and 825.6 (aromatic C-H stretching) cm^−1^ was also examined for Cu- and Cr- treated cells, respectively.

**Table 3:** The frequencies (cm^−1^) and assignments of FTIR bands for fungal cells of *T. brevicompactum* QYCD-6 before and after treatment with aqueous solutions of 50 mg/L Cu, Cr, Cd, Pb, Zn and metal mix.

Untreated cells	Loaded cells						Suggested assignment
	Cu	Cr	Cd	Pb	Zn	Metal mix	
3424.0	3385.1	3373.9	3357.9	3423.6	3362.2	3373.6	O-H and N-H stretching vibrations
2924.9	2924.1	2925.3	2926.5	2924.9	2926.0	2926.3	C-H asymmetric stretching
—	2853.3	—	—	2853.3	—	—	C-H symmetric stretching
—	2426.1	2426.1	—	—	—	—	P-H stretching
—	1744.5		—	—	—	—	C=O stretching of ester
1652.1	1655.1	1655.1	1655.1	1651.9	1655.3	1651.8	C=O stretching and N-H deformation (amide I region)
1552.3	1548.5	1551.1	1546.8	—	1543.3	1546.7	N-H bending in amide II and C-N stretching in -CO-NH-
1400.9	1384.3	1384.7	1406.2	1382.8	1405.5	1406.6	carboxylate group
1154.7	1155.5	1155.4	1152.8	1153.7	1152.2	1150.3	Phosphate and sulfide groups
1074.4	1077.5	1074.5	1079.9	1077.4	1080.5	1079.5	Phosphate and sulfide groups
1034.3	1038.7	1032.6	1033.8	1038.6	1034.5	1050.2	C-C, C=C, C-O-C, C-O-P of saccharides
—	—	825.6	—	—	—	—	Aromatic C-H stretching
562.6	577.1	563.5	578.2	579.1	580.3	574.8	Nitro compounds and disulfide groups

The changes in vibrational frequencies examined by FTIR analysis on the surface of metal-treated fungus supported the involvement of biosorption for metal removal^1^. The biosorption mechanisms were mainly based on physicochemical interactions between the metal ions and the functional groups^32–34^. The participation of negatively charged groups (e.g. carbonyl, phosphoryl and hydroxyl groups) of the fungal cells suggested interactions with positively metal ions for heavy metal removal^1,35^. The type of functional groups present depended on the fungal species^36^. For examples, Pb removal by *B. bassiana* isolated by Gola *et al*.^37^ primarily involved protein (N-H), carbohydrate, fatty acids esters and protein amide groups, while the uptake of Cd by *Phanerochaete chrysosporium* was attributed to hydroxyl, carboxylic and amino functional groups^38^. Hydroxyl, ethers, amines/amides, carboxylic acid/carboxylate and phosphatidate groups of *Penicillium chrysogenum* CS1 were involved in Cr and Pb adsorption^39^, whereas hydroxyl, amides, carboxyl, and sulfhydryl groups of *Pleurotus ostreatus* ISS-1 were drawn into Pb adsorption^40^.

### Solid-state NMR analysis

**Figure 4:**
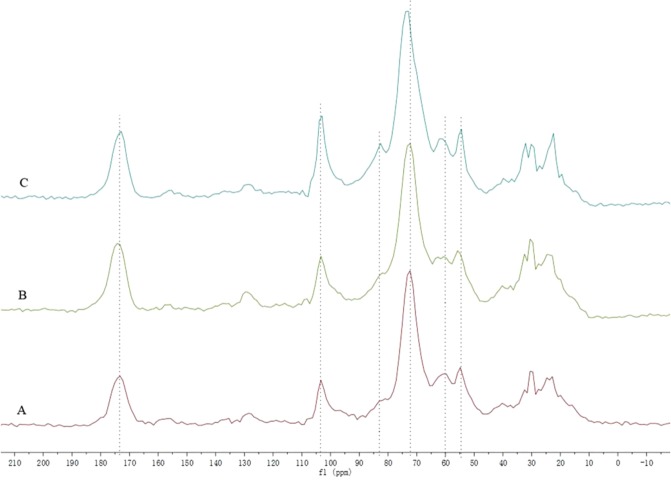
Solid-state ^13^C CP/TOSS NMR spectra of *T. brevicompactum* QYCD-6. (**A**) In absence of heavy metal; (**B**) at 50 mg L^−1^ Pb; (**C**) at 50 mg L^−1^ Cu.

Solid-state NMR gives the possible information on functional group present on the cell surface^41^ and their interaction with the heavy metals, which support and complement the findings from the FTIR analysis^42^. The solid-state ^13^C CP/TOSS NMR of fungal biomass grown in the control as well as in the presence of heavy metal Pb and Cu were obtained (Fig. 4). The signals at 54–70 ppm, 70–103 ppm and 103 ppm respectively corresponded to the carbons adjacent to a heteroatom, the carbons adjacent to oxygen in carbohydrates, and heteroaromatic (C-1) atoms^42,43^, which suggested the groups of hydroxyl or amino were involved. Figure 4 displayed the numbers of absorption peaks (54–103 ppm region and 103 ppm) were shifted in the presence of heavy metal Pb and Cu, which indicated the negatively charged groups (e.g. hydroxyl and amino groups) participated in interactions with positively charged metal ions. In addition, the signal at approximately 173 ppm was assigned to carboxylic group^39^. Treatment with Cu and Pb caused peaks to a little shift at 173.4 in ^13^C solid-state NMR, which indicated that the carboxylic group was involved in the interactions with heavy metals.

## Conclusion

In the present investigation, an earthworm gut fungus *T. brevicompactum* QYCD-6 was isolated from *P. tschiliensis*. Compared with other metals [Cu(II), Cr(VI), Cd(II) and Zn(II)], the fungus had the highest tolerance to Pb(II) with the MIC value of 1600 mg L^−1^ on PD medium. Moderate (45.9%) and the highest removal (97.5%) by the fungus were observed for multimetal and Pb(II), respectively. TEM demonstrated the cellular changes of fungus induced by heavy metals. FTIR and solid-state NMR indicated specific functional groups contributing towards metal uptake. To the best of our knowledge, there are no studies on the metal removal by the intestinal microorganisms of earthworms and hence the strain *T. brevicompactum* QYCD-6 may be a promising candidate for the remediation of heavy-metal polluted sites.

## Supplementary information

Supplementary Information.
