# Supplementary material for: Multiple heavy metal tolerance and removal by an earthworm gut fungus Trichoderma brevicompactum QYCD-6
Source: Sci Rep. 2020 Apr 24;10:6940. doi: 10.1038/s41598-020-63813-y (PMC7181882; doi:10.1038/s41598-020-63813-y)
Supplement: Supplementary file 1 — Supplementary Information. [file 41598_2020_63813_MOESM1_ESM.docx]

**Supplementary material**

**Multiple heavy metal tolerance and removal by an earthworm gut fungus *Trichoderma brevicompactum* QYCD-6**

Ding Zhang^1,†^, Caiping Yin^1,†^, Naeem Abbas^1^, Zhenchuan Mao^2,^*, Yinglao Zhang^1,^*

^1^ *School of Life Sciences, Anhui Agricultural University, Hefei 230036, PR China*

^2^ Institute of Vegetables and Flowers, Chinese Academy of Agricultural Sciences, Beijing 100081, China

* Corresponding authors.

**E-mail address:**

[maozhenchuan@caas.cn](mailto:maozhenchuan@caas.cn) (Z.C. Mao), Tel.: +86-010-82109545;

zhangyl@ahau.edu.cn (Y. L. Zhang), Tel.: +86-551-6578-6129

†These authors have contributed equally to this work.


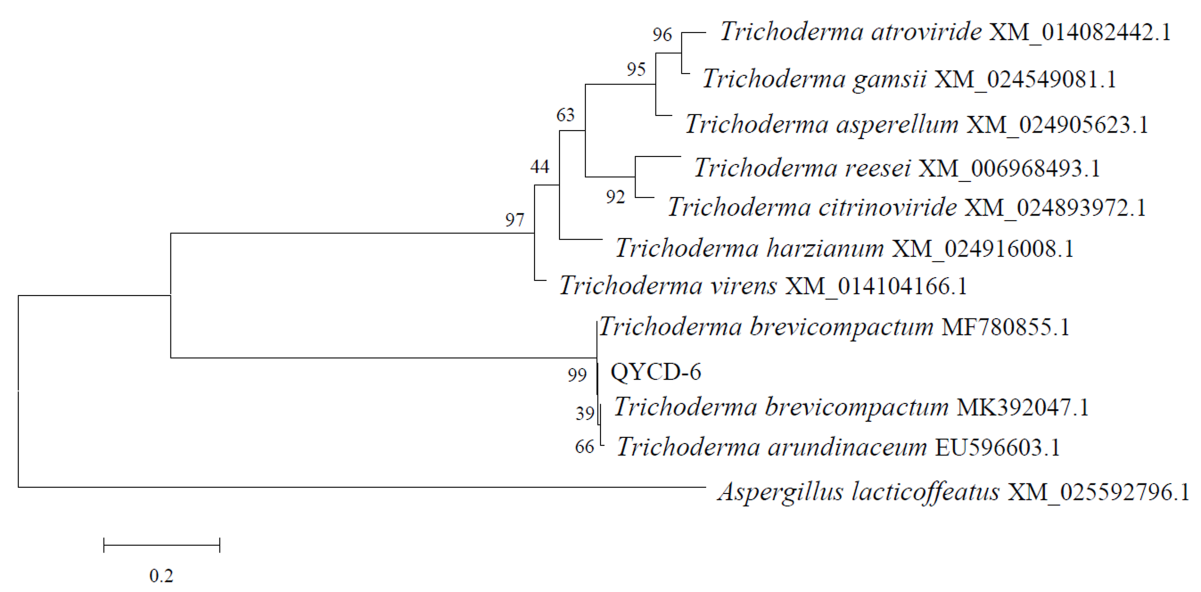


**Fig. S1.** Phylogenetic tree of the fungus QYCD-6 based on the 5.8S rDNA sequences.


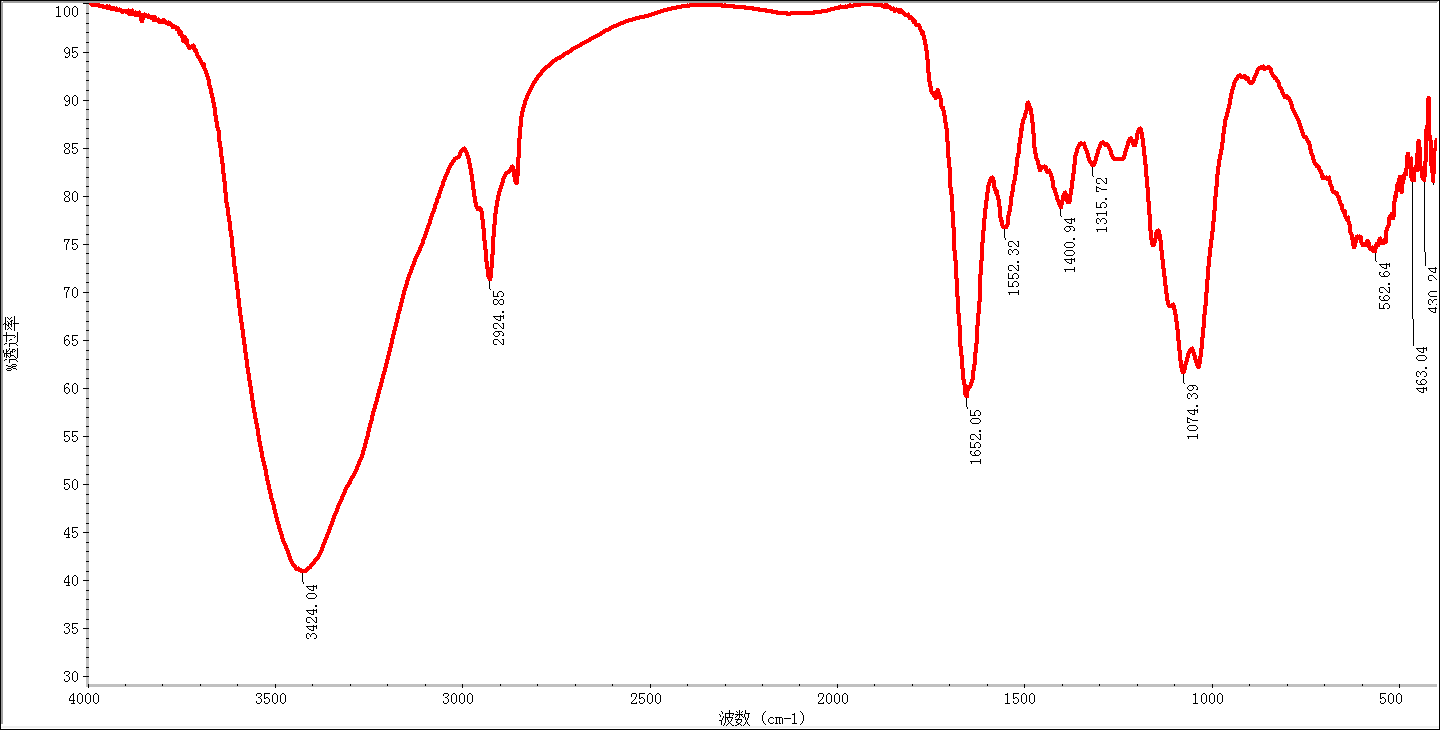


A


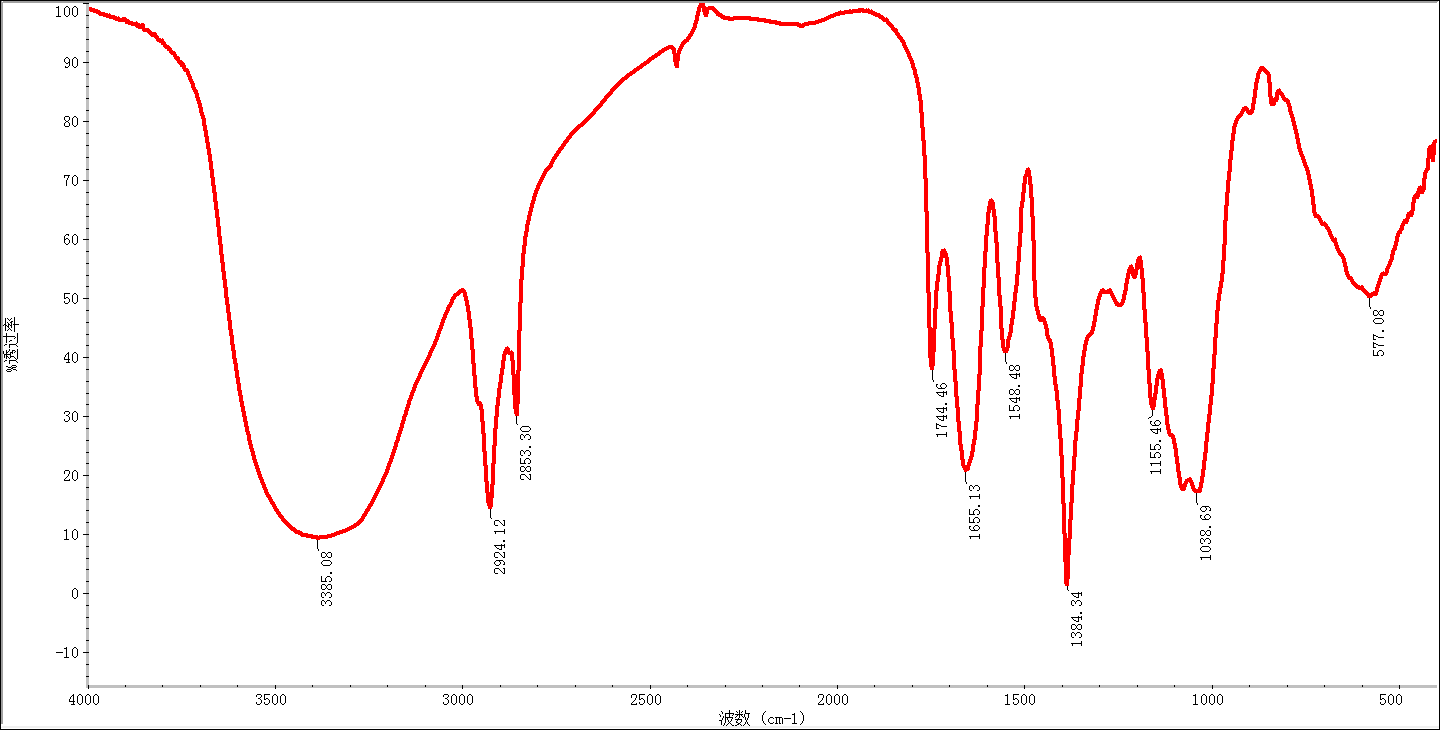


B


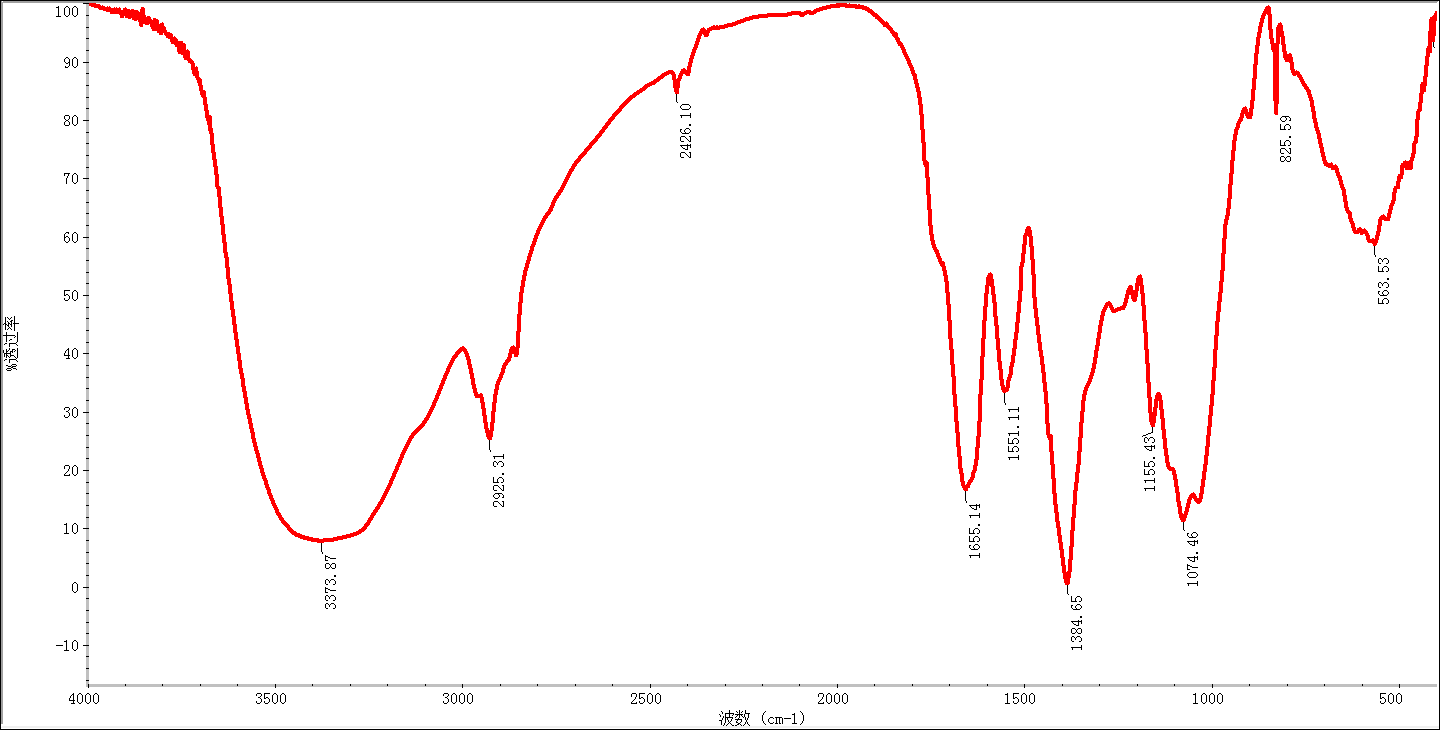


C


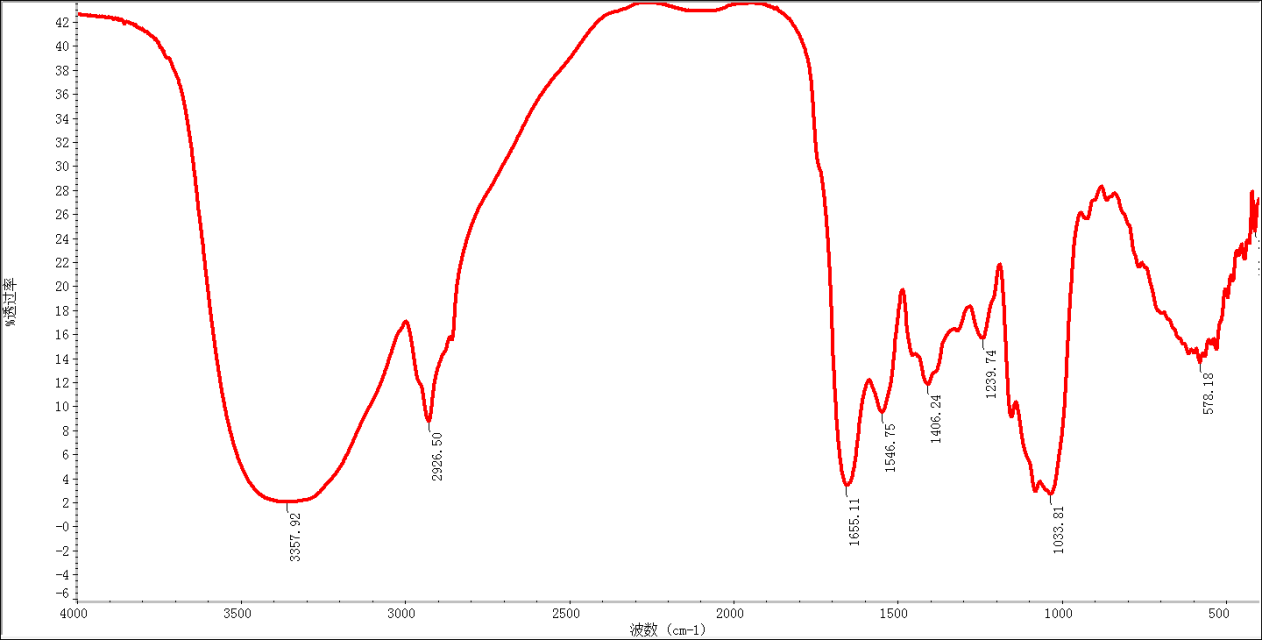


D


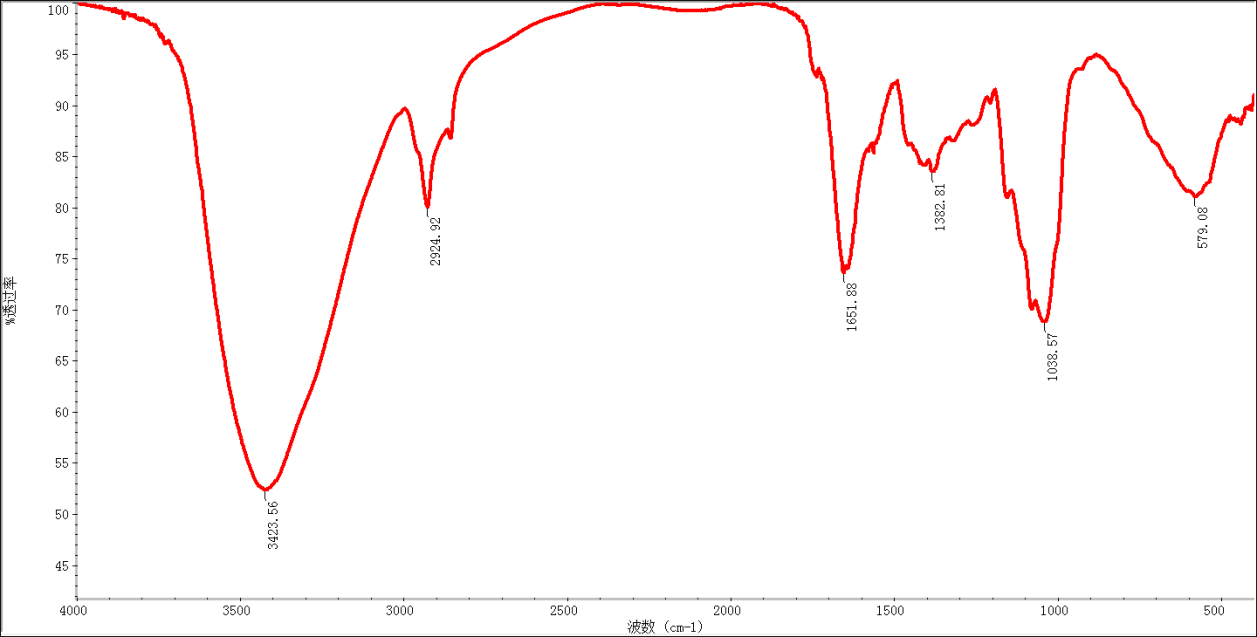


E


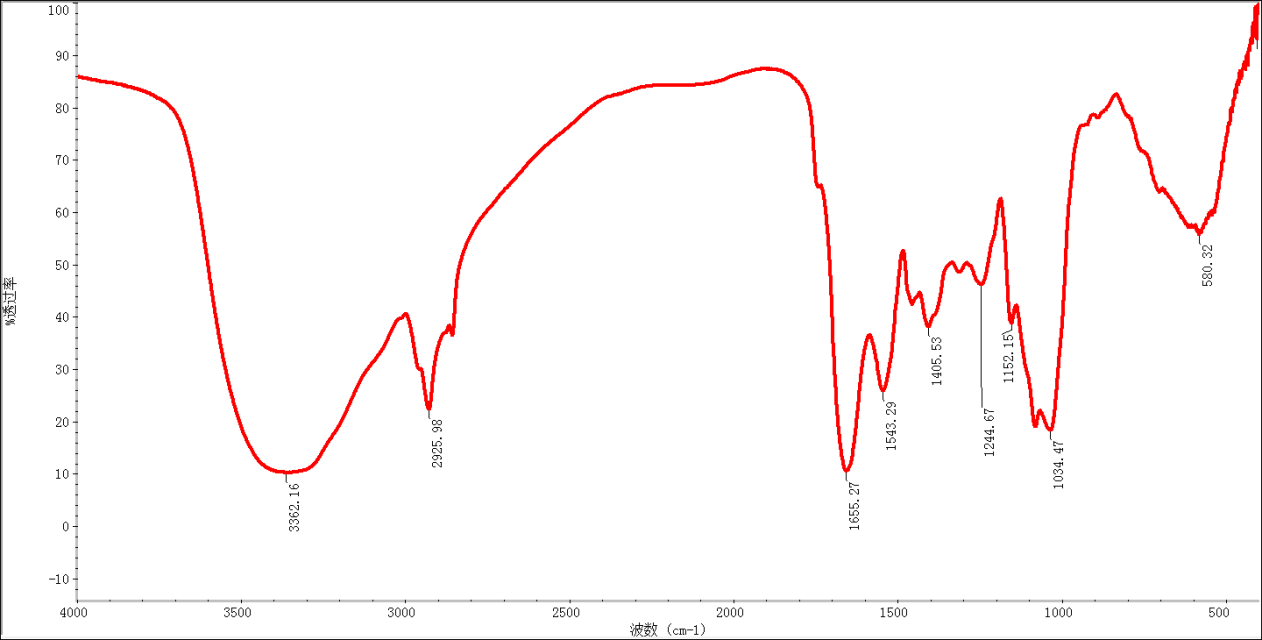


F


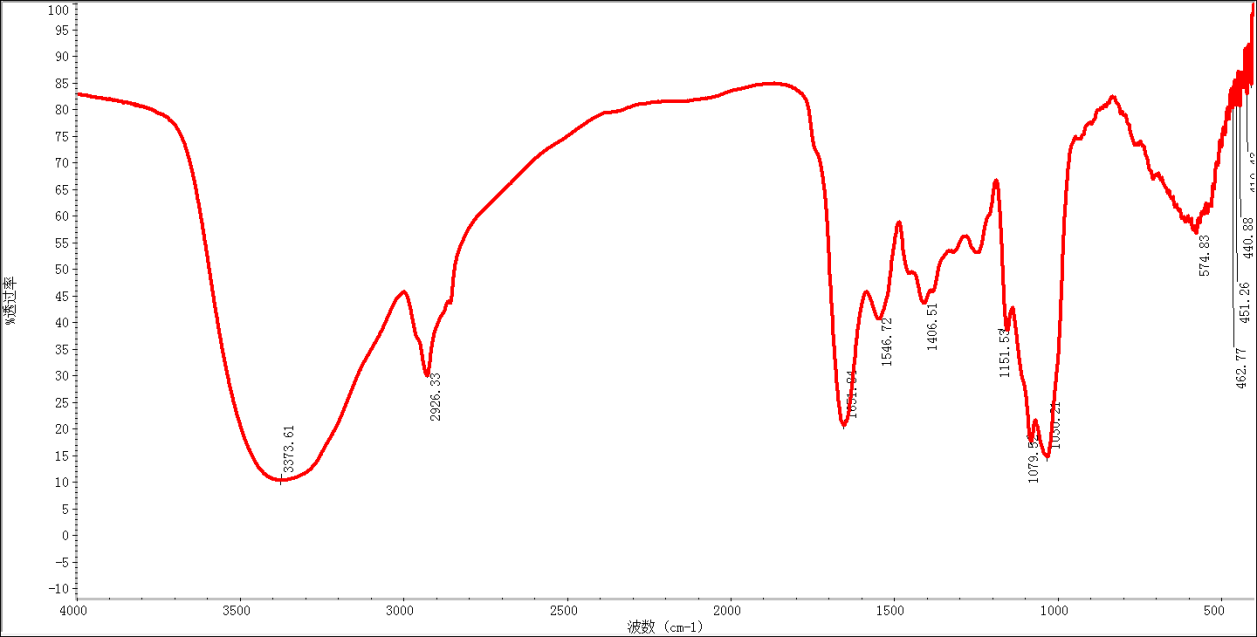


G

Figure S2. FTIR spectra of *T. brevicompactum*. (A) In absence of Heavy Metal; (B) at 50 mg L^-1^ Cu; (C) at 50 mg L^-1^ Cr; (D) at 50 mg L^-1^ Cd; (E) at 50 mg L^-1^ Pb; (F) at 50 mg L^-1^ Zn; (G) at 50 mg L^-1^ Multi Metal Mix.
